# Species‐specific habitat preferences do not shape the structure of a crested newt hybrid zone (*Triturus cristatus* x *T. carnifex*)

**DOI:** 10.1002/ece3.5683

**Published:** 2019-10-02

**Authors:** Zdeněk Mačát, Martin Rulík, Daniel Jablonski, Antonín Reiter, Lenka Jeřábková, Stanislav Rada, Peter Mikulíček

**Affiliations:** ^1^ Department of Ecology and Environmental Sciences Palacky University Olomouc Czech Republic; ^2^ Department of Zoology Comenius University Bratislava Slovakia; ^3^ South Moravian Museum in Znojmo Znojmo Czech Republic; ^4^ Nature Conservation Agency of the Czech Republic Praha Czech Republic

**Keywords:** amphibia, hybridization, microsatellites, mtDNA, reproductive isolation barrier, Salamandridae

## Abstract

Reproductive isolation barriers maintain the integrity of species by preventing interspecific gene flow. They involve temporal, habitat or behavioral isolation acting before fertilization, and postzygotic isolation manifested as hybrid mortality or sterility. One of the approaches of how to study reproductive isolation barriers is through the analysis of hybrid zones. In this paper, we describe the structure of a hybrid zone between two crested newt species (*Triturus cristatus* and *T. carnifex*) in the southern part of the Czech Republic using morphological, microsatellite, and mitochondrial (mtDNA) markers. Specifically, we tested the hypothesis that the structure of the hybrid zone is maintained by species‐specific habitat preferences. Comparing the genetic structure of populations with geographical and ecological parameters, we found that the hybrid zone was structured primarily geographically, with *T. cristatus*‐like populations occurring in the northeast and *T. carnifex*‐like populations in the southwest. Despite *T. cristatus* tending to occur in deeper ponds and *T. carnifex* on localities with more shading, the effect of both ecological parameters on the structure of the zone was minimal. Next, we corroborated that *T. carnifex* individuals and some hybrids possess mtDNA of *T. dobrogicus*, whose nuclear background was not detected in the studied hybrid zone. Hybridization between *T. carnifex* and *T. dobrogicus* (resulting in unidirectional mtDNA introgression) had to predate subsequent formation of the hybrid zone between *T. cristatus* and *T. carnifex*. Populations of crested newts in the southern part of the Czech Republic thus represent a genetic mosaic of nuclear and mitochondrial genomes of three species.

## Graphical abstract

In this paper, we describe the structure of the hybrid zone between two crested newt species (*Triturus cristatus* and *T. carnifex*) in the southern part of the Czech Republic using morphological, microsatellite, and mitochondrial (mtDNA) markers. Specifically, we tested the hypothesis that the structure of the hybrid zone is maintained by species‐specific habitat preferences. Contrary to our expectations, we found that the hybrid zone was structured only geographically but not ecologically, with *T. cristatus*‐like populations occurring in the northeast and *T. carnifex*‐like populations in the southwest.

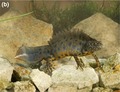

## 1 INTRODUCTION

Reproductive isolation barriers prevent hybridization and gene flow between biological species (e.g., Coyne & Orr, 2004). They act either before (prezygotic) or after (postzygotic) fertilization. Prezygotic barriers include temporal and habitat isolation when species reproduce at another time or in another preferred habitat. Species may also exhibit species‐specific mating behavior, incompatibility of their reproductive organs or gametes. Postzygotic isolation is associated with incompatibilities between genomes of hybridizing taxa, manifested by increased mortality or reduced fertility of first and subsequent generation hybrids. The study of prezygotic and postzygotic barriers is therefore crucial to understanding the origin and maintenance of species in nature.

One of the approaches for understanding the evolution of reproductive isolation barriers, interspecific gene flow (introgression) and mechanisms of speciation is through the analysis of hybrid zones. These transition zones are relatively narrow regions of admixed genotypes originating when two diverged populations (e.g., species, subspecies, or chromosomal races) come into contact, mate, and produce hybrid progeny (Hewitt, 2001). They are characterized by abrupt changes in species‐specific traits (alleles in the case of genetic markers) along a geographic transect or cline (Barton, 1983, 2001). The width of the cline increases with the dispersal rate of the parental genotypes and decreases with selection against intrinsically incompatible hybrid genotypes (endogenous selection). The dispersal of parental genotypes into the hybrid zone center is the main source of nonrandom associations among loci (linkage disequilibrium). Linkage disequilibrium generates interactions among selected loci that increase the effective selection of any one locus. When hybrid zones occur in ecotones, where both parental species are adapted to different habitats, the steepness of the cline and its width might be, moreover, influenced by selection against genotypes in the alternative habitat (exogenous selection; Arias et al., 2008; Barton & Gale, 1993; Shurtliff, Murphy, & Matocq, 2013; Yanchukov et al., 2006). Habitat isolation between the parental species may thus limit interspecific gene flow and determine the nature of species boundaries. In this study, we focused on hybridization between newt species belonging to the *Triturus cristatus* superspecies group in order to determine whether habitat preferences shape the structure of the hybrid zone and thus can be involved in species isolation. We tested whether there is an association between the genotypic composition of populations and the type of aquatic habitats which newts use for reproduction.

The *Triturus cristatus* superspecies group includes seven currently recognized closely related species widespread in Europe, Asia Minor, and the Caucasian‐–Caspian region (Arntzen, Üzüm, Ajduković, Ivanović, & Wielstra, 2018; Wielstra & Arntzen, 2011; Wielstra & Artnzen, 2016; Wielstra, McCartney‐Melstad, Arntzen, Butlin, & Shaffer, 2019). Three species are found in Central Europe: *T. cristatus* (Laurenti, 1768), *T. dobrogicus* (Kiritzescu, 1903), and *T. carnifex* (Laurenti, 1768). While *T. cristatus* is widely distributed from the British Isles to the Ural Mountains, *T. dobrogicus* is restricted to the lowlands of the middle and lower Danube. *Triturus carnifex* occurs in the Apennine Peninsula and a north Adriatic part of the Balkans, from whence it spreads its range into Central Europe (Arntzen, 2003; Wielstra & Arntzen, 2011; Wielstra, Sillero, Vörös, & Arntzen, 2014).

*Triturus cristatus*, *T. dobrogicus,* and *T. carnifex* were recorded in southern Moravia, a historical part of the Czech Republic, where their hybridization was later documented (Mikulíček, Horák, Zavadil, Kautman, & Piálek, 2012; Piálek, Zavadil, & Valíčková, 2000; Reiter & Hanák, 2000; Zavadil, Piálek, & Klepsch, 1994). The most complex population structure was observed in the Znojmo region, where *T. cristatus* comes into contact with *T. carnifex*. The majority of *T. carnifex* individuals and their hybrids with *T. cristatus* possess an introgressed mitochondrial genome of *T. dobrogicus* (Mikulíček et al., 2012), the nearest populations of which are, however, located 40 km to the east of the Znojmo transect. The hybrid zone in southern Moravia is several kilometers wide (the shortest straight geographic distance between *T. cristatus* and *T. carnifex* populations is ca 15 km), but the mechanisms preventing hybridization between the species and maintaining the width of the zone are unknown.

In the present study, we focus on species‐specific habitat preferences between *T. cristatus* and *T. carnifex* in a hybrid zone situated in southern Moravia. We assume that if the hybrid zone were shaped by ecological preferences of the crested newt species, genetic composition of their populations should correlate with specific habitat characteristics. Specifically, we aim (a) to describe the structure of the hybrid zone based on morphological, microsatellite, and mitochondrial markers using more extensive sampling than in a previous study (Mikulíček et al., 2012) and (b) to find out whether there is an association between genotypic composition of populations and aquatic (reproductive) habitats of newts.

## 2 MATERIAL AND METHODS

### 2.1 Sampling

In this study, we focused on the region of southern Moravia (Czech Republic), where a contact zone between three crested newt species has been documented (Mikulíček et al., 2012). Individuals (*n* = 300) were caught on 38 sampling sites during the breeding season between the years 2010–2015 (Table 1). Funnel collapsible nylon traps with bait (chicken liver) were used for catching, following the methodology of Bock, Hennig, and Steinfartz, (2009). A toenail clip was removed and stored in 96% ethanol.

**Table 1:** Sampling sited, their abbreviations, structure ID, coordinates, and number of crested newts analyzed for mtDNA (*n*_mtDNA_), nuclear markers – microsatellites (*n*_mtsats_) and GenBank accession number

Locality name	Acronym	Structure ID	Latitude	Longitude	Altitude m a.s.l.	n_mtDNA_	n_msats_	GenBank accession number
Popice	POP	12	48.819°N	16.007°E	318	3	14	MN394483‐5
Tasovice	TAS	11	48.820°N	16.153°E	205	3	12	MN394482, MN394505, MN394523
Podmolí – Pustý ryb.	POPR	23	48.843°N	15.935°E	420	3	5	MN394499, MN394500‐1
Podmolí – tůně	POTU	25	48.843°N	15.937°E	424	3	5	MN394503, MN394506‐7
Podmolí – strouha	POST	7	48.847°N	15.943°E	409	1	2	MN394478
Mašovice – střelnice	MAST	13	48.847°N	15.977°E	387	1	1	MN394486
Mašovice – lom	MALO	14	48.857°N	15.987°E	358	0	18	–
Hradiště	HRKA	36	48.860°N	16.006°E	351	1	1	MN394528
Červený rybníček	CERY	37	48.861°N	16.025°E	327	0	1	–
Lukov	LUK	28	48.866°N	15.892°E	443	3	11	MN394511‐3
Čížov – Malý ryb.	CIMR	27	48.874°N	15.869°E	413	3	8	MN394508‐10
Citonice	CIT	41	48.876°N	15.961°E	367	3	3	MN394535‐7
Braitava	BRAJ	16	48.885°N	15.794°E	444	2	9	MN394487‐8
Vranov	VRSL	34	48.885°N	15.841°E	317	1	2	MN394527
Čížov ‐ tůně	CIZT	10	48.885°N	15.884°E	412	2	19	MN394480‐1
Únanov	UNAN	43	48.889°N	16.051°E	311	0	8	–
Čížov – Lesní ryb.	CILE	6	48.890°N	15.877°E	411	3	23	MN394475‐7
Onšov	ONS	15	48.906°N	15.847°E	464	1	5	MN394490
Žerůtky	ZER	5	48.908°N	15.968°E	374	1	9	MN394474
Čekál	CEK	4	48.935°N	15.949°E	372	1	16	MN394531
Chvalatice	CHVA	35	48.936°N	15.744°E	443	2	2	MN394524‐5
Hostěradice	HOST	18	48.949°N	16.277°E	265	3	6	MN394491‐2, MN394495
Bojanovice – Veský ryb.	BOVR	38	48.950°N	15.979°E	349	2	2	MN394532‐3
Bojanovice – U Huberta	UHTU	33	48.953°N	15.997°E	356	2	12	MN394521‐2
Mikulovice	MIKU	9	48.956°N	16.117°E	351	0	13	–
Šanderka	SAN	17	48.960°N	15.727°E	486	1	1	MN394532
Vevčice	VEV	8	48.960°N	16.026°E	334	2	9	MN394479, MN394530
Zblovice	ZBLO	42	48.965°N	15.707°E	482	0	9	–
Jevišovice	JEV	24	48.979°N	15.964°E	390	2	6	MN394502, MN394529
Trstěnice	TRST	29	49.000°N	16.175°E	325	1	5	MN394514
Čermákovice	CERM	32	49.024°N	16.198°E	371	2	17	MN394519‐20
Horní Kounice – cihelna	HKCI	30	49.025°N	16.137°E	369	3	6	MN394515‐6
Hostim – u Kyničky	HOKY	26	49.028°N	15.926°E	429	2	6	MN394504, MN394526
Moravský Krumlov – Sáňkova louka	MKSL	22	49.028°N	16.353°E	346	1	4	MN394493‐4
Horní Kounice – Valovo j.	HKVJ	31	49.035°N	16.155°E	347	2	7	MN394517‐8
Moravský Krumlov – tůně u Kulatého palouku	MKKP	20	49.039°N	16.390°E	310	0	20	–
Moravský Krumlov – Polesí	MKPO	19	49.042°N	16.360°E	390	2	5	MN394498
Jamolice	JAM	21	49.079°N	16.227°E	386	2	7	MN394496‐7
Horní Slatina	HOSL	44	49.097°N	15.558°E	524	0	5	–
Třebětice	TREB	40	49.048°N	15.532°E	481	0	6	–
Nová Říše	NORI	39	49.145°N	15.567°E	531	0	10	–
Řečice	RECI	3	49.141°N	15.371°E	545	0	3	–
Plachta	PLA	2	50.031°N	14.727°E	370	2	10	MN394540‐1
Matena	MATE	1	45.971°N	14.498°E	295	2	15	MN394538‐9

### 2.2 DNA markers and laboratory techniques

Population genetic structure and the degree of hybridization between the crested newts were inferred by two types of genetic markers: bi‐parentally inherited nuclear microsatellites and maternally inherited mtDNA. Sixty‐six individuals from the Moravian hybrid zone were analyzed for mtDNA. We amplified the >1,400 bp‐long portion of mtDNA comprising the complete *ND2* gene, five subsequent transfer RNA (tRNAs) genes and the light‐strand replication origin using primers (L3780, H5018) and protocol following Krupa et al. (2002). The final analyzed stretch contained a 620 bp‐long fragment of *ND2*. The sequencing was performed by Macrogen Inc. (Seoul, South Korea and Amsterdam, Netherlands; http://www.macrogen.com). The novel sequences were deposited in GenBank under accession numbers: MN394474–MN394541. The *ND2* fragment was aligned using the Clustal W algorithm (Thompson, Ling, & Grustein, 1994) as implemented in BioEdit (Hall, 1999). Alignments were checked by eye and low‐quality ends were trimmed. Ambiguously aligned regions/gaps were ignored for the subsequent analysis. No stop codons were detected when the sequences were translated using the vertebrate mitochondrial genetic code in the program DnaSP 5.10. We used a network approach (Posada & Crandall, 2001) to infer interindividual/species relationships. A haplotype network for three species of *Triturus* was constructed using PopArt 1.7 (http://popart.otago.ac.nz; French et al., 2014) and the implemented median‐joining algorithm.

Seven microsatellite loci (Krupa et al., 2002) were amplified in all sampled individuals. Additionally, reference (allopatric) populations from Slovenia (*T. carnifex*, *n* = 15), the southern part of Slovakia (*T. dobrogicus*, *n* = 20), and the northern part of the Czech Republic (*T. cristatus*, *n* = 25) were analyzed to establish allele frequencies of microsatellite loci in the parental species.

Microsatellites were amplified in two multiplex PCRs (multiplex 1: *Tcri13*, *Tcri 29*, *Tcri 36*, *Tcri 46*; multiplex 2: *Tcri 27*, *Tcri 35*, *Tcri 43*) using primers labeled with fluorochromes NED, PET, VIC, and FAM, and the Qiagen multiplex PCR kit (Qiagen). Thermal profiles for both multiplex amplifications consisted of 15 min initial denaturation at 95°C followed by 35 cycles of 30 s at 94°C, 90 s at 60°C, 60 s at 72°C, and 30 min at 60°C.

Two model‐based methods were used to estimate the proportion of admixture from multilocus genotype data applying a Bayesian approach implemented in the programs Structure 2.3.3 (Pritchard, Stephens, & Donnelly, 2000) and Geneland 3.1.4 (Guillot, Estoup, Mortier, & Cosson, 2005; Guillot, Mortier, & Estoup, 2005). These programs assign individuals into *K* clusters with minimized Hardy–Weinberg and linkage disequilibria. In a first procedure in Structure, all individuals were assigned to the inferred clusters (from *K* = 1 to *K* = 10) without any a priori population information. In a second procedure, a priori population information for individuals from reference populations was used. Individuals from populations in or close to a presumable contact zone were assigned to the clusters without using any a priori information. To evaluate convergence and to estimate optimal genetic clustering, five replicates were run for each *K* value using an admixture and uncorrelated allele model. All Structure analyses were based on runs of 10^6^ iterations, following a burn‐in period of 10^4^ iterations. The number of populations that best fitted the data set was defined by the Δ*K* method (Evanno, Regnaut, & Goudet, 2005), as implemented in Structure Harvester (Earl & vonHoldt, 2012). For each individual, we calculated the *q* values which define the proportion of an individual's genome that originated from cluster *K*. The *q* values were calculated also for populations as a mean of individuals' *q* values.

In Geneland, we analyzed only samples from the presumable contact zone (southern part of the Czech Republic). First, we ran analyses with *K* free to vary, to infer the optimal value of this parameter. We ran the analysis 10 times to verify the consistency of the results, with the following parameters: 500,000 MCMC iterations, maximum rate of Poisson process fixed to 100, zero uncertainty of coordinates, minimum and maximum *K* 1 to 10, maximum number of nuclei in the Poisson–Voronoi tessellation fixed to 300, and null allele and uncorrelated allele models. All 10 replicates revealed the maximum posteriori estimate of *K* = 2. Then, we ran the MCMC 10 times with *K* fixed to 2 and the same parameter settings. The run with the highest log probability was chosen for postprocess analyses. The posterior probability of population membership for each pixel of the spatial domain and for each individual was then computed with 500 pixels along the *X* and *Y* axes. The modal population of each individual, maps of population membership, and maps of probability of population membership were finally computed.

To estimate the level of genetic diversity, we applied the program GenAlEx 6.5 (Peakall & Smouse, 2012) for calculation of the number of alleles, the coefficient of inbreeding (*F*_IS_), and observed (*H*_O_) and expected (*H*_E_) heterozygosity. These parameters were calculated for each population under study and then for three groups in the contact zone, that is “pure” populations of *T. cristatus*, “pure” populations of *T. carnifex* and hybrid populations. Assignment of populations to these groups was based on an admixture proportion of each individual (parameter* q* according to Pritchard et al., 2000, i.e., the proportion of an individual's genome that originates from the *T. cristatus* and *T. carnifex* cluster) averaged for a population. Populations were considered as hybrid when they possessed more than 20% of introgressed microsatellite alleles (i.e., average population *q* value was ≤0.8).

### 2.3 Morphological characteristics and comparison with microsatellite markers

Morphological differences between *T. cristatus* and *T. carnifex* are conspicuous. They differ in the body habitus, and the relative length of the trunk and legs (Arntzen & Wallis, 1999). These differences might be expressed as the “Wolterstorff” index, or WI (Wolterstorff, 1923) which is defined as the ratio between forelimb length (Pa) and interlimb distance (LiE; WI = 100 × Pa/LiE). The values of WI increase from *T. cristatus* to *T. carnifex*.

Morphological characteristics were obtained from anesthetized newts (0.8% solution of 2‐phenoxyethanol) using a dial‐caliper (with an accuracy of 0.5 mm). We measured the following characteristics: *L*—body length, Lcd—tail length, Ltot—total body length, Lc1—jaw length, Lc2—head length, Ltc—head width, Pa—front limb (on both sides of the body), Pp—hind limb (on both sides of the body), LiE—interlimb distance (on both sides of the body). All 12 characteristics were measured by the same caliper and same person.

We used RDA (Redundancy analysis) to find out which morphological characteristics discriminate between both newt species. RDA models were constructed in Canoco 5 (TerBraak & Šmilauer, 2012). The *q* values calculated in Structure based on microsatellite loci entered the analysis as response variables (log transformed), morphological characteristics as explanatory variables, weight, and sex as covariables. Two characteristics, that is, interlimb distances measured on both sides of the body, were removed from the RDA analysis because of their collinearity (mutual and with some other variables). The statistical significance of the model, first axis, and individual morphological factors were tested by Monte Carlo permutation (999 repetitions).

Then, we tested if WI discriminates between *T. cristatus* and *T. carnifex* and thus is a reliable morphological marker for species delimitation in the studied hybrid zone. Males and females were analyzed separately. First, we carried out Spearman correlation between the *q* values from Structure which define the proportion of individual's genome that originated from the *T. cristatus* and *T. carnifex* genome, respectively, and WI values. Spearman correlation was calculated using the SPSS program (IBM Corp., 2015). Second, we estimated the percentage of individuals misclassified using the WI. Again, we used the *q* values from Structure to assign individuals to a particular species. An individual was assigned to *T. cristatus* or *T. carnifex* when the proportion of its genome (the *q* value) originating from *T. cristatus* or *T. carnifex* cluster was equal or higher than 0.8.

### 2.4 Association between genotypic composition of populations and habitat characteristics

In order to test preferences of particular species to specific habitats, we compared genotypic composition of populations with environmental variables. For each population, we calculated average *q* values from Structure, which were entered (after the log transformation) to the analysis as response variables.

Seven habitat characteristics of ponds where crested newts reproduced, plus geographic coordinates (latitude and longitude), were recorded in April 2014. These habitat characteristics are relevant for the distribution of crested newts as was found in previous studies (Maletzky, Kyek, & Goldschmid, 2007). Specifically, we measured pond area (m^2^), maximum depth in three classes (<30 cm, 30–100 cm, >100 cm), fish presence or absence, origin of pond (natural or artificial), and presence or absence of human use. We also estimated density of submerged vegetation (in 25%‐classes) and proportion of shade (in 25%‐classes). These characteristics (including latitude and longitude) were tested to collinearity and then entered into the analysis as explanatory variables.

The RDA model was constructed in Canoco 5 (TerBraak & Šmilauer, 2012). Statistical significance of the model, first axis, and individual habitat preference factors were tested by Monte Carlo permutation (999 repetitions).

## 3 RESULTS

### 3.1 Mitochondrial DNA

**Figure 1:**
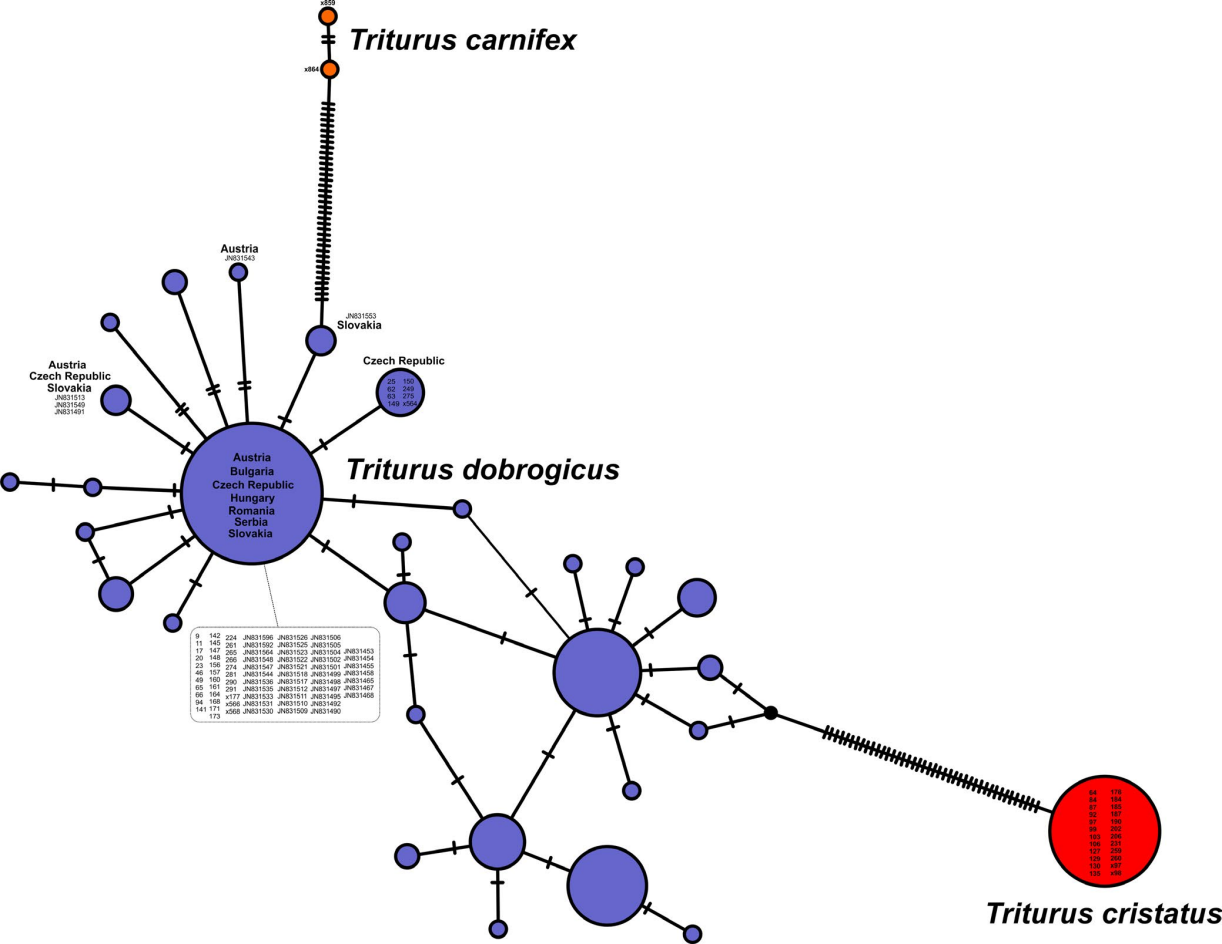
Phylogenetic haplotype network in crested newts from the Moravian hybrid zone sequenced for ND2 fragment. The size of the symbols is proportional to the observed haplotype frequency in *Triturus cristatus* (red), *T. dobrogicus* (purple), and *T. carnifex* (orange). Numbers indicate either the ID of individuals sequenced in this study or GenBank entries (JNxx) originally published by Vörös et al. (2016)

Out of 66 newts from the Moravian hybrid zone sequenced for *ND2* fragment, 24 individuals possessed one *T. cristatus*‐specific haplotype and 42 possessed two *T. dobrogicus*‐specific haplotypes. *Triturus cristatus*‐ and *T. dobrogicus*‐specific haplotypes occurred in the northern and southern part of the hybrid zone, respectively (Figure 1).

In order to find out the origin of *T. dobrogicus* mtDNA, we compared haplotypes from the Znojmo region with haplotypes from the whole range of *T. dobrogicus* (Vörös, Mikulíček, Major, Recuero, & Arntzen, 2016). Haplotypes of *T*. *dobrogicus* from the study area clustered with haplotypes vastly distributed in the western Pannonian Basin, including northwestern Austria, southeastern Czech Republic and western Slovakia, that is, the nearest areas where *T. dobrogicus* is distributed (Figure 1). One haplotype of *T*. *dobrogicus* was unique for the study area but diverged by just two mutation steps from the vastly distributed haplotype.

### 3.2 Microsatellite markers

**Figure 2:**
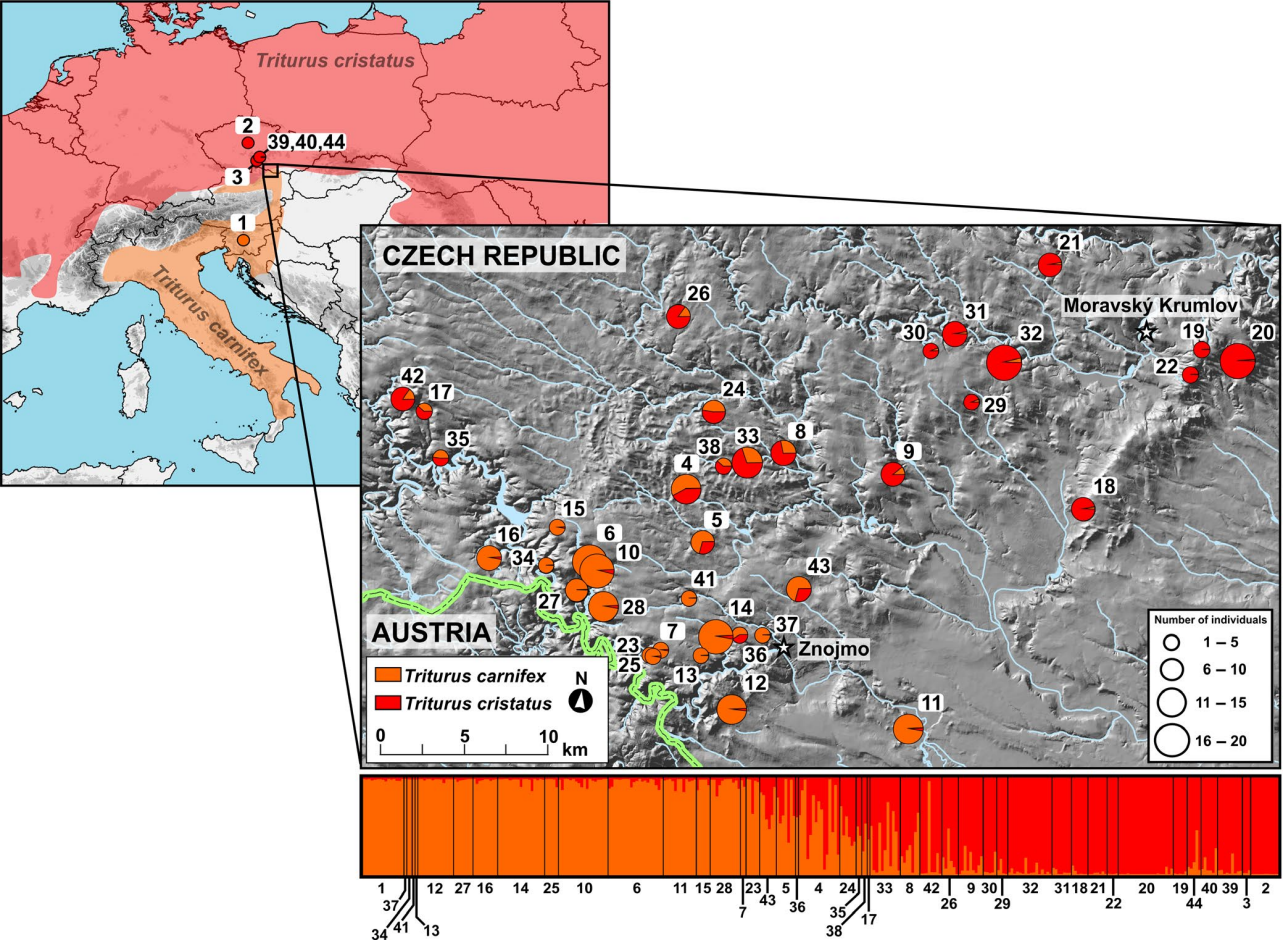
Geographical distribution and proportion of admixture (parameter *q* according to Structure) between *T. cristatus* (red) and *T. carnifex* (orange) in south Moravia, Czech Republic estimated on the basis of microsatellite data

Out of the seven microsatellite loci, two (*Tcri29* and *Tcri35*) revealed many missing genotypes and difficult‐to‐interpret patterns in a fragmentary analysis. Therefore, these loci were excluded from population genetic analyses. Structure Harvester using the Δ*K* method estimated the most likely number of clusters for *K* = 2 (Δ*K* = 960.5). Cluster 1 and cluster 2 corresponded to *T. cristatus* and *T. carnifex*, respectively. The second highest optimal cluster number, for *K* = 6, had much lower probability (Δ*K* = 32.7). Newts from reference populations were assigned to the correct Structure clusters (corresponding to the parental species) with an average probability *q* = 0.978 for *T. carnifex* (the only reference locality Matena) and *q* = 0.837–0.986 for *T. cristatus* populations (Table 2). Specimens originating from the contact zone were either assigned to the parental species or showed mixed ancestry between *T. cristatus* × *T. carnifex* (Table 2, Figure 2). The hybrid zone forms a sharp and geographically structured cline, with *T. cristatus*‐like populations located in the northeast and *T. carnifex*‐like populations in the southwest.

**Table 2:** Summary of population genetic variation of crested newts based on microsatellites

Site	*N* (±*SE*)	*H*_O_ (±*SE*)	*H*_E_ (±*SE*)	*q‐cri*	*q‐car*
MATE^a^	14.4 (±0.4)	0.573 (±0.156)	0.595 (±0.143)	.021	.978
CERY	1.0 (±0.0)	0.600 (±0.245)	0.600 (±0.245)	.017	.983
VRSL	1.8 (±0.2)	0.300 (±0.200)	0.433 (±0.194)	.018	.981
CITO	1.8 (±0.49)	0.800 (±0.200)	0.667 (±0.184)	.019	.981
MAST	0.8 (±0.2)	0.400 (±0.245)	0.400 (±0.245)	.021	.979
POP	11.4 (±1.86)	0.332 (±0.088)	0.503 (±0.078)	.021	.978
CIMR	6.6 (±0.245)	0.571 (±0.092)	0.611 (±0.051)	.023	.976
BRAJ	8.0 (±0.775)	0.491 (±0.084)	0.561 (±0.103)	.027	.972
MALO	15.2 (±2.154)	0.431 (±0.063)	0.591 (±0.130)	.030	.969
POTU	4.4 (±0.6)	0.400 (±0.141)	0.516 (±0.113)	.033	.967
CIZT	17.2 (±0.86)	0.542 (±0.092)	0.623 (±0.053)	.039	.960
CLES	20.0 (±1.643)	0.425 (±0.083)	0. 569 (±0.083)	.043	.956
TAS	11.2 (±0.8)	0.358 (±0.139)	0.472 (±0.134)	.044	.955
ONS	4.2 (±0.8)	0.480 (±0.150)	0.462 (±0.127)	.052	.947
LUK	9.2 (±0.663)	0.432 (±0.116)	0.504 (±0.089)	.053	.946
POST	2.0 (±0.0)	0.400 (±0.100)	0.467 (±0.133)	.059	.941
POPR	4.8 (±0.2)	0.450 (±0.112)	0.604 (±0.088)	.097	.903
UNAN	6.4 (±0.245)	0.762 (±0.095)	0.604 (±0.065)	.292	.707
ZER	6.8 (±0.735)	0.596 (±0.085)	0.677 (±0.061)	.307	.692
HRKA	0.8 (±0.2)	0.600 (±0.245)	0.600 (±0.245)	.406	.594
CEKL	15.2 (±0.374)	0.537 (±0.037)	0.698 (±0.061)	.420	.579
JEV	6.0 (±0.0)	0.767 (±0.125)	0.815 (±0.017)	.539	.460
CHVA	2.0 (±0.0)	0.700 (±0.122)	0.733 (±0.100)	.546	.453
BOVE	2.0 (±0.0)	0.800 (±0.122)	0.800 (±0.082)	.611	.389
SAND	0.8 (±0.2)	0.600 (±0.245)	0.600 (±0.245)	.630	.370
UHTU	11.4 (±0.245)	0.662 (±0.064)	0.752 (±0.040)	.687	.312
VEV	7.6 (±0.6)	0.670 (±0.103)	0.709 (±0.038)	.713	.286
ZBLO	8.4 (±0.245)	0.500 (±0.018)	0.692 (±0.032)	.832	.167
HOKY	4.6 (±0.4)	0.720 (±0.102)	0.729 (±0.057)	.835	.164
MIKU	10.4 (±1.077)	0.457 (±0.093)	0.598 (±0.105)	.884	.115
HKCI	5.2 (±0.374)	0.653 (±0.160)	0.633 (±0.076)	.927	.073
TRS	4.4 (±0.245)	0.400 (±0.113)	0.633 (±0.076)	.935	.064
CERM	16.0 (±0.548)	0.593 (±0.088)	0.654 (±0.068)	.954	.045
HKVJ	7.0 (±0.0)	0.600 (±0.079)	0.666 (±0.034)	.964	.035
HOST	6.0 (±0.0)	0.500 (±0.118)	0.521 (±0.123)	.966	.033
JAM	6.4 (±0.6)	0.421 (±0.130)	0.582 (±0.115)	.977	.022
MKPO	5.0 (±0.0)	0.480 (±0.136)	0.440 (±0.130)	.978	.021
MKKP	19.4 (±0.245)	0.629 (±0.084)	0.577 (±0.075)	.979	.020
MKSL	4.0 (±0.0)	0.500 (±0.079)	0.586 (±0.069)	.984	.015
HOSL^a^	4.6 (±0.245)	0.610 (±0.040)	0.705 (±0.089)	.837	.162
TREB^a^	6.0 (±0.0)	0.767 (±0.113)	0.715 (±0.105)	.889	.110
NORI^a^	9.0 (±0.316)	0.641 (±0.084)	0.606 (±0.030)	.944	.056
RECI^a^	3.0 (±0.0)	0.800 (±0.082)	0.720 (±0.080)	.967	.033
PLA^a^	10.0 (±0.0)	0.580 (±0.174)	0.488 (±0.094)	.986	.013

Abbreviations: *H*_E_, expected heterozygosity; *H*_O_, observed heterozygosity; *N*, average number of analyzed individuals; *q‐cri*, *q‐car*, probability of each individual to belong to one of the two inferred clusters corresponding to the parental species *T. cristatus* and *T. carnifex* (mean *q* values per population).

a Localities out of study region.

**Figure 3:**
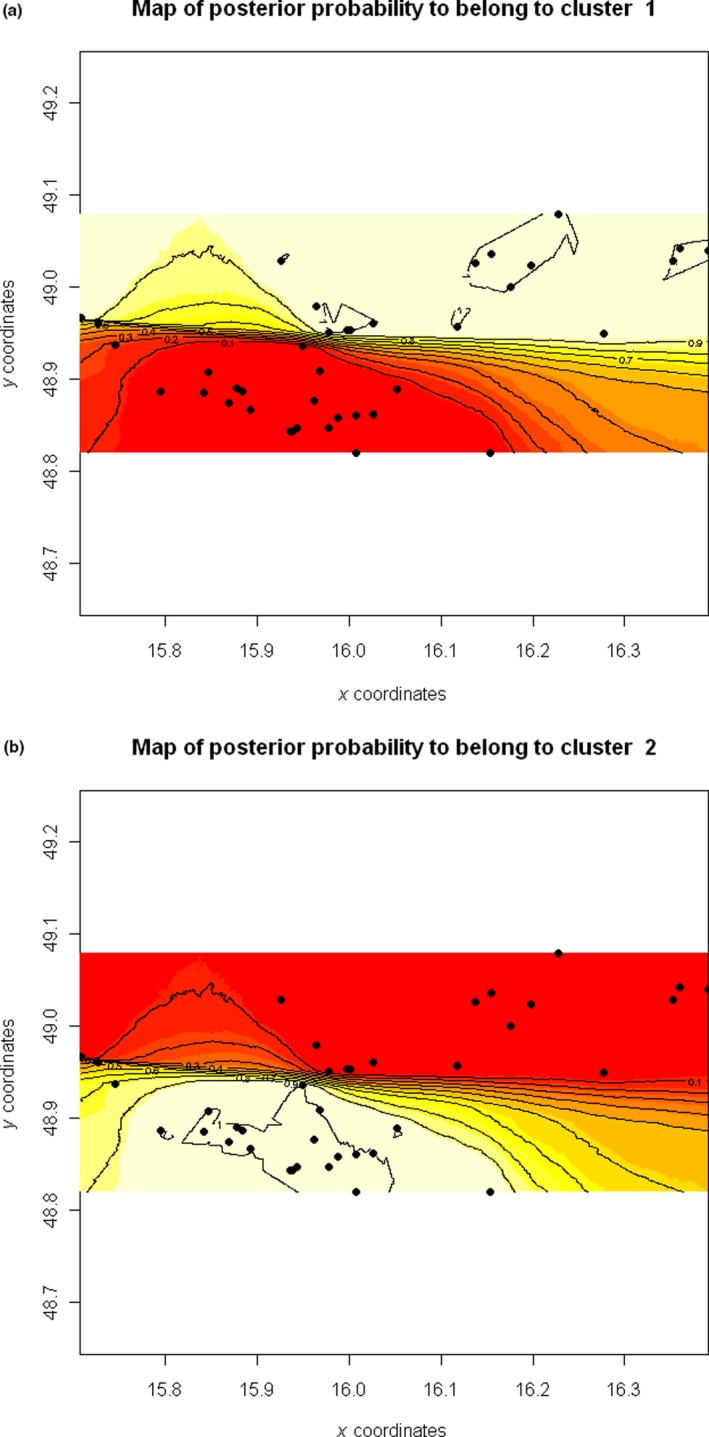
Maps of posterior probability of population membership using Geneland. The northerly distributed cluster 1 corresponds to *T. cristatus*, the southerly distributed cluster 2 corresponds to *T. carnifex*. A hybrid zone is shown as a sharp cline. The axes indicate the longitude (*X* coordinates) and latitude (*Y* coordinates)

Geneland corroborated results from Structure and revealed two clusters corresponding to the hybridizing parental species (Table 2). Cluster 1 and cluster 2 corresponded to *T. carnifex*‐like and *T. cristatus*‐like populations, respectively (Figure 3). The transition of genotypes from one cluster into the other is abrupt, showing that the contact zone between both species is a narrow region where species hybridize.

Parameters of genetic diversity for each population are summarized in Table 2. “Pure” populations of the parental species and hybrid populations from the contact zone revealed a comparable level of genetic diversity (Table 1).

### 3.3 Morphological characters and their comparison with microsatellite markers

Partial RDA analysis explained 28.60% of variation in samples, the first axis explained 28.39% of variability, the second axis 0.21%, and the third axis explained 65.38% of variation. Test on the first axis (pseudo‐*F* = 94.4, *p* = .001) and on all axes (pseudo‐*F* = 10.6, *p* = .001) confirmed the statistical significance of the RDA model. Simple term effects of individual morphological variables (Table 3) suggest which of them discriminate between the species. Out of 12 morphological characteristics only four (Ltc, L, LiE1, and LiE2) did not significantly discriminate between *T. cristatus* and *T. carnifex*.

**Table 3:** Simple term effects of morphological characteristics entering the partial RDA model with genotypic composition of populations as response variables

Name	Explains %	pseudo‐*F*	*p*
Pp R (mm)	13.8	39.4	.001
Pp L (mm)	10.2	27.8	.001
Lc 2 (mm)	9.9	27.2	.001
Pa R (mm)	5.5	14.4	.001
Lc 1 (mm)	4.5	11.7	.001
Pa L (mm)	4.2	10.7	.002
Lcd (mm)	3.0	7.7	.008
Ltot (mm)	2.3	5.8	.017
Ltc (mm)	1.5	3.8	.051
*L* (mm)	1.2	2.9	.085

Note

Two characteristics (interlimb distances on both sides of the body, LiE1, and LiE2) were removed from the RDA analysis because of their collinearity.

Abbreviations: *L*, body length; Lc1, jaw length; Lc2, head length; Lcd, tail length; Ltc, head width; Ltot, total body length; Pa, front limb (on both sides of the body); Pp, hind limb (on both sides of the body).

Comparison between morphological index (WI) and microsatellite genotypes was used for designation of reliability of WI for species determination. Correlation between the *q* values from Structure which define the proportion of individual's genome that originated from the *T. cristatus* and *T. carnifex* genome, respectively, and WI values were significant in both males and females (Spearman correlation; males, *r* = 0.310, *p* = .001; females, r = 0.254, *p* = .004).

Nevertheless, the percentage of misclassification based on the WI was high. Out of 89 males assigned to *T. cristatus* on the basis of the WI (WI < 64), only 40 were assigned to this species according to microsatellite markers (*q* ≥ 0.8). Out of 32 males assigned to *T. carnifex* on the basis of the WI (WI > 64), 22 were assigned to this species according to microsatellites. Similarly, out of 96 females assigned to *T. cristatus* on the basis of the WI (WI < 54), only 40 were assigned to this species using microsatellites. Out of 33 females assigned to *T. carnifex* on the basis of the WI (WI > 54), 22 were assigned to this species genetically.

This means that 55.1% and 58.3% of *T. cristatus* males and females were misclassified using WI. In *T. carnifex,* the percentage of misclassification in males and females was 31.3% and 33.3%, respectively.

### 3.4 Association between genotypic composition of populations and habitat characteristics

**Figure 4:**
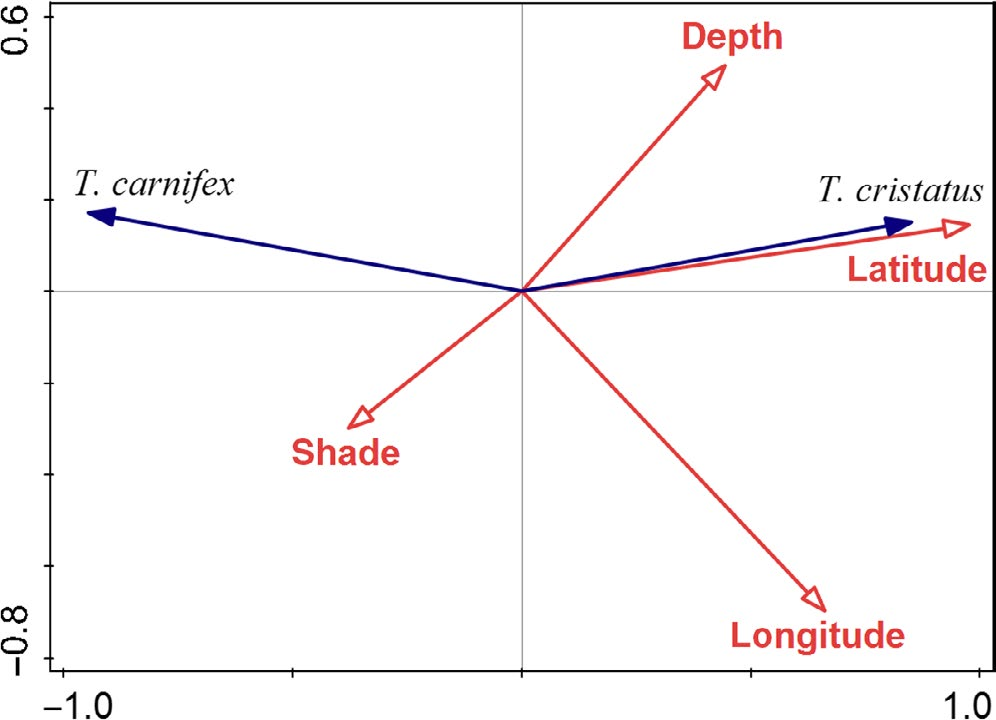
Ordination diagram of RDA model with genotypic composition of populations as response variables and habitat characteristics as explanatory variables. Latitude was the most important factor, explaining 76.2% of variability

Genotypic composition of populations based on microsatellites (*q* values per population in Table 2) was compared with habitat characteristics. Within all geographical and environmental variables, only latitude, longitude, depth, and shading were statistically significant and thus were included into the final model. The model explained 82.5% of variation in the data. The most important factor was latitude, explaining 76.2% of variability; followed by longitude, explaining 5.1%. Effects of depth and shading were minimal but their simple term effects were statistically significant. *Triturus cristatus* tended to occur in deeper ponds, while *T. carnifex* occurred on localities with more shading (Figure 4).

## 4 DISCUSSION

Crested newts are widely distributed amphibians, with parapatric distributions which come into contact and hybridize in Central Europe and the Balkans (Arntzen et al., 2018; Arntzen, Wielstra, & Wallis, 2014; Maletzky et al., 2008; Wielstra, Burke, Butlin, & Arntzen, 2017; Wielstra, Burke, Butlin, Avcıc, et al., 2017). The rate of hybridization and introgression have been studied in several transects between different crested newt species but the role of species‐specific habitat preferences in the architecture of hybrid zones has not been studied in detail. In this paper, we extended our previous study (Mikulíček et al., 2012) and corroborated the occurrence of the hybrid zone between *T. cristatus* and *T. carnifex* in the southern part of the Czech Republic (Znojmo region). We also corroborated that *T. carnifex* and hybrid individuals in the studied zone possess the mitochondrial genome of *T. dobrogicus*, a species which is distributed in the lowlands of the middle and lower Danube River but has not been documented in the Znojmo region. Next, we found that the Znojmo transect is structured primarily geographically but not ecologically. *Triturus cristatus* and *T. carnifex* do not reveal marked species‐specific habitat preferences, and their hybrid zone is unlikely to be maintained by exogenous selection. Finally, we evaluated the use of morphological characteristics for species recognition and found that most of them significantly discriminated between “pure” *T. cristatus* and *carnifex* individuals. Comparison of the Wolterstorff index (WI) and microsatellite genotypes, however, revealed that this index is not reliable for species identification in the studied hybrid zone.

### 4.1 Reliability of morphological markers for species delimitation

The morphological variability of crested newts was thoroughly studied during the last century (Arntzen & Wallis, 1994, 1999; Kalezić, Džukić, Stamenković, & Crnobrnja, 1990; Şova, 1973; Wolterstorff, 1923). Herpetologists have identified morphological traits which could be used for recognition of individual crested newt species. In general, *T. cristatus* is a medium‐built newt with shorter legs. In contrast, *T. carnifex* has a medium to heavy‐built body and longer legs, while the interlimb distance in both species is the same. Also, coloration is different, *T. cristatus* shows distinctive white stippling along their sides/legs, and an orange belly with a variable pattern of black spots. By contrast, *T. carnifex* shows little or no white stippling, and a yellow belly with large, round, ill‐defined greyish to black spots. Females often have a yellow vertebral stripe (e.g., Fahrbach & Gerlach, 2018). Hybrids are often intermediate in their phenotypic characters (Arntzen & Wallis, 1994; Brede, Thorpe, Arntzen, & Langton, 2000).

In this study, we tested whether morphological characteristics discriminate between *T. cristatus* and *T. carnifex*. Even though eight out of ten morphological traits significantly discriminated between the “pure” *T. cristatus* and *T. carnifex* individuals, the Wolterstorff index (WI) as a traditional marker used for crested newt delimitation (Arntzen & Wallis, 1999; Wolterstorff, 1923) failed in the studied hybrid zone. More than a third of *T. carnifex* and a half of *T. cristatus* individuals were misclassified according to the WI. This finding corresponds to Arntzen and Wallis (1999), who showed 31% misclassification of newts using WI. On the contrary, Brede et al. (2000), who morphologically evaluated hybrids between introduced *T. carnifex* and native *T. cristatus* in UK, were able to recognize parental species and F1 hybrids based on morphological traits. In the Czech hybrid populations, however, the existence of different hybrid categories (mainly backcross hybrids with one or another parental species and F2 hybrids) and extensive introgression (Mikulíček et al., 2012) prevent the use of WI in species identification.

### 4.2 Species‐specific habitat preferences

One of the characteristics that defines species in nature is their ecological differentiation involving habitat preferences. In phylogenetically closely related species, ecological differentiation, however, could be very subtle. We evaluated the habitat preferences of *T. cristatus* and *T. carnifex* comparing the habitat features of the studied localities with the genotypic composition of newt populations.

Habitat suitability for crested newts has been studied by several authors, but most of the research was focused just to one species (e.g., Blab & Blab, 1981; Harper, Downie, & McNeill, 2018; Oldham, Keeble, Swan, & Jeffcote, 2000; Unglaub, Steinfartz, Drechsler, & Schmidt, 2015). For instance, Maletzky et al. (2007) evaluated habitat features of crested newt populations in northwestern Austria, where *T. cristatus* and *T. carnifex* come into contact and hybridize (Maletzky et al., 2008). The authors however did not discriminate between the hybridizing species. They found that shading and density of submerged vegetation had significant effects on pond occupancy (Maletzky et al., 2007). Blab and Blab (1981), besides shading and density of submerged vegetation, identified also pond area as the relevant characteristic for the abundance of crested newts. Other authors showed other key habitat features, including water chemistry and the structure of terrestrial habitat (Cooke, Cooke, & Sparks, 1994; Harper et al., 2018; Sztatecsny, Jehle, Schmidt, & Arntzen, 2004). Arntzen and Wallis (1999) pointed out habitat preferences between *T. cristatus* and *T. carnifex* in the Basin of Geneva (Switzerland and adjacent France) where *T. carnifex* was introduced. While *T. cristatus* preferred ponds containing an abundance of aquatic vegetation, *T. carnifex* thrived in disturbed quarries with little or no vegetation. Another study focusing on hybridization between both species reveals an expansion of the introduced *T. carnifex* at the expense of the native *T. cristatus* in the Netherlands and links this displacement (among others) to the wider ecological amplitude of *T. carnifex* and its greater resistance to disturbed aquatic habitats (Meiling, Arntzen, van Delft, & Wielstra, 2015).

According to our observations, it is clear that both temporary and permanent water bodies with water levels from just a few centimeters to several meters and from water surface ranging from a few square meters to many hectares may provide suitable aquatic habitat for the studied crested newts. Our main aim however was to identify the differences in habitat preferences between both species. *Triturus cristatus* tended to occur in deeper ponds, while *T. carnifex* occurred in ponds with more shading, but the effect of both characteristics was minimal. Thus, we can postulate that *T. cristatus* and *T. carnifex* do not show marked species‐specific habitat preferences in the southern part of the Czech Republic. However, it is important to note that we measured only selected habitat characteristics, and therefore, we cannot exclude that other habitat features, including water chemistry and the structure of terrestrial habitat, may discriminate between the species.

### 4.3 Structure of the hybrid zone

The absence of marked species‐specific habitat preferences indicates that the structure of the hybrid zone between *T. cristatus* and *T. carnifex* in the Znojmo region is not shaped ecologically. The zone is structured primarily geographically with *cristatus*‐like genotypes in the northeast and *carnifex*‐like genotypes in the southwest. We assume that this pattern might have been established when the two species came into contact during their recolonization of Central Europe after the last glaciation. Pleistocene glaciations led to retraction of ranges of many European species which survived in refugia (i.e., regions with suitable climatic and ecological conditions) located southerly. At the end of the Pleistocene, when ice‐sheets retreated and climatic conditions became more hospitable, species dispersed out of refugia and colonized the newly available areas further north. In the case of the studied species, *T. cristatus* had a glacial refugium in the Carpathians (Wielstra, Babik, & Arntzen, 2015) and thus had to colonize the Znojmo region postglacially from the west. *Triturus carnifex* had to reach Central Europe from the south, while its glacial refugia were probably located in the Adriatic region (Canestrelli, Sacco, & Nascetti, 2011). When the two newt species came into contact, they established a secondary hybrid zone which is currently geographically structured from the northeast to the southwest (Hewitt, 2001, 2011).

This scenario is very likely also in the case of hybridizing crested newts—with one remarkable note. All *T. carnifex* individuals and some hybrids with *T. cristatus* possess mtDNA of *T. dobrogicus*, a species geographically restricted to the lowlands of the middle and lower Danube, and which is not currently distributed in the Znojmo region. It follows that *T. carnifex* and *T. dobrogicus* had to meet and hybridize in the past, despite the present distribution of both species having a ca 40 km gap (Piálek et al., 2000; Reiter & Hanák, 2000; Zavadil et al., 1994). The occurrence of *T. dobrogicus* mtDNA haplotypes specific for the western Pannonian Basin in the Znojmo hybrid zone indicates that historical hybridization between *T. carnifex* and *T. dobrogicus* might have taken place east of the Alps, probably in the western edge of the Pannonian Basin (Vörös & Artzen, 2010). Hybridization was then followed by unidirectional mtDNA introgression and the spread of *T. carnifex* individuals possessing *dobrogicus* mtDNA to the areas of the present‐day hybridization with *T. cristatus*. If this scenario was applied in the studied crested newt system, this would be another example when an alien (introgressed) organellar genome “surfed the wave” of a range expansion on a “borrowed (nuclear) board” (Klopfstein, Currat, & Excoffier, 2006; Neiva, Pearson, Valero, & Serrão, 2010).

When we compare our *T. cristatus* x *T. carnifex* transect with other crested newt hybrid zones, we can postulate that the transition zone in Znojmo is not shaped by ecological differentiation of the hybridizing species. It seems that parental genotypes do not show adaptation to alternative environments where exogenous selection could play a significant role. This is in stark contrast with a *T. cristatus* x *T. marmoratus* hybrid zone in southwestern France where the hybridizing species are ecologically well differentiated. While *T. marmoratus* occupies mainly forested and hilly areas, *T. cristatus* is distributed in open and flat country (Schoorl & Zuiderwijk, 1980; Visser, Leeuw, Zuiderwijk, & Arntzen, 2016). Both species also reveal up to twice higher genetic divergence in comparison with *T. cristatus* and *T. carnifex*, a bimodal type of the hybrid zone with limited hybridization and introgression caused by strong postzygotic genomic incompatibilities (Arntzen, Jehle, Bardakci, Burke, & Wallis, 2009).

Ecological differentiation can play a significant role also in zones where *T. dobrogicus* hybridizes with other crested newt species (*T. cristatus*, *T. carnifex*, *T. ivanbureschi*, and *T. macedonicus*; for details see Arntzen et al., 2014). While *T. dobrogicus* is restricted to lowlands, other crested newt species occur mainly in hilly areas and mountains. Moreover, *T. dobrogicus* is better adapted to a more aquatic mode of life by its slender, elongated body and shorter limbs (Arntzen, Bugter, Cogalniceanu, & Wallis, 1997; Arntzen & Wallis, 1999). It can be assumed that elevation together with relief and temperature are the most important ecological factors preventing hybridization between *T. dobrogicus* and other crested newt species (Arntzen et al., 2014; Mikulíček et al., 2012). Such marked ecological differences however are not known between *T. cristatus* and *T. carnifex*, nor did our study reveal any significant habitat preferences. The structure of the *T. cristatus* x *T. carnifex* hybrid zone in the Znojmo transect is thus probably shaped by endogenous selection associated with postzygotic genomic incompatibilities. For instance, artificial male hybrids between *T. cristatus* × *T. carnifex* are fertile and able to produce other generations of hybrids, but these hybrids reveal higher mortality, disturbed meiosis, and production of dysfunctional gametes (Arntzen et al., 2014; Callan & Spurway, 1951; MacGregor et al., 1990 and references herein). The structure of the hybrid zone between *T. cristatus* and *T. carnifex* thus might be maintained by a balance between the dispersal of parental genotypes into the center of the zone and selection against hybrids; that is, two mechanisms playing an important role in the tension zone model (Barton & Hewitt, 1985; Key, 1968; Macholán et al., 2007; Szymura & Barton, 1986, 1991).

The width of the *T. cristatus* and *T. carnifex* hybrid zone in southern Moravia was estimated by Mikulíček et al. (2012) at ca 15 km. The shortest straight geographic distance between *T. cristatus*‐like (locality 18) and *T. carnifex*‐like (locality 11) populations in this study (covering more populations and individuals) was ca 17 km. The fact that hybridization and interspecific gene flow are restricted to a relatively narrow transect depends (besides selection against hybrids) on the limited dispersal abilities of newts (Arntzen & Wallis, 1991; Meiling et al., 2015) and their strong site fidelity (Mori et al., 2017).

Hybrid zones are characterized by abrupt changes in species‐specific allelic frequencies along a cline, the width of which depends on the strength of selection and dispersal of parental genotypes. Loci under selection, revealing a sharp cline and limited interspecific introgression, influence the rate of neutral introgression. Hybrid zones thus might be seen as barriers preventing gene flow between the hybridizing species. The strength of such a barrier can be determined not only by endogenous selection associated with incompatibilities between parental genomes, but also by exogenous selection acting when parental genotypes, adapted to alternative habitats, are selected in the wrong environment. In the present study, we did not find marked species‐specific habitat preferences between the crested newts *T. cristatus* and *T. carnifex*, which indicates that adaptation to alternative environments and exogenous selection do not play a significant role in the structure of the hybrid zone. On the contrary, the zone is structured geographically. Spatial distribution of the parental and hybrid genotypes likely reflects the postglacial colonization of Central Europe from two different directions. On this colonization “road,” the gene pool of *T. carnifex* was enriched by the mitochondrial genome of a third species, *T. dobrogicus*. Newt populations in southern Moravia thus represent a genetic mosaic of nuclear and mitochondrial genomes of three crested newt species historically or currently hybridizing.

## CONFLICT OF INTERESTS

The authors declare that they have no competing interests.

## AUTHOR CONTRIBUTIONS

The authors have made the following declarations about their contributions: ZM, MR, and PM conceived and designed the study. ZM, AR, and LJ collected data in field. ZM, DJ, SR, and PM analyzed the data. ZM, DJ, and PM wrote the manuscript. All authors participated in the scientific discussions, revised the manuscript, and read and approved the final manuscript.

## Data Availability

DNA sequences: Genbank accessions—MN394474–MN394541.

## References

[ece35683-bib-0001] Arias, C. F. , Munoz, A. G. , Jiggins, C. D. , Mavarez, J. , Bermingham, E. , & Linares, M. (2008). A hybrid zone provides evidence for incipient ecological speciation in *Heliconius* butterflies. Molecular Ecology, 17, 4699–4712. 10.1111/j.1365-294X.2008.03934.x 18828780

[ece35683-bib-0002] Arntzen, J. W. (2003). *Triturus cristatus* Superspezies – Kammolch‐Artenkreis In Grossenbacher K., & Thiesmeier B. (Eds.), Handbuch der Reptilien und Amphibien Europas, Band 4/IIA, Schwanzlurche (Urodela) IIA, Salamandridae II: Triturus I (pp. 421–514). Wiebelsheim, Germany: AULA‐Verlag.

[ece35683-bib-0003] Arntzen, J. W. , Bugter, R. J. F. , Cogalniceanu, D. , & Wallis, G. P. (1997). The distribution and conservation status of the Danube crested newt, *Triturus dobrogicus* . Amphibia‐Reptilia, 18, 133–142. 10.1163/156853897X00026

[ece35683-bib-0004] Arntzen, J. W. , Jehle, R. , Bardakci, F. , Burke, T. , & Wallis, G. P. (2009). Asymmetric viability of reciprocal‐cross hybrids between crested and marbled newts (*Triturus cristatus*) and *T. marmoratus* . Evolution, 63, 1191–1202. 10.1111/j.1558-5646.2009.00611.x 19154385

[ece35683-bib-0005] Arntzen, J. W. , Üzüm, N. , Ajduković, M. D. , Ivanović, A. , & Wielstra, B. (2018). Absence of heterosis in hybrid crested newts. PeerJ, 6, e5317 10.7717/peerj.5317 30065885 PMC6063215

[ece35683-bib-0006] Arntzen, J. W. , & Wallis, G. P. (1991). Restricted gene flow in a moving hybrid zone of the newts *Triturus cristatus* and *T. marmoratus* in western France. Evolution, 45, 805–826. 28564049 10.1111/j.1558-5646.1991.tb04352.x

[ece35683-bib-0007] Arntzen, J. W. , & Wallis, G. P. (1994). The ‘WOLTERSTORFF index’ and its value to the taxonomy of the Crested Newt superspecies. Abhandlungen und Berichte für Naturkunde, 17, 57–66.

[ece35683-bib-0008] Arntzen, J. W. , & Wallis, G. P. (1999). Geographic variation and taxonomy of crested newt (*Triturus cristatus* superspecies): Morphological and mitochondrial DNA data. Contribution to Zoology, 68, 181–203.

[ece35683-bib-0009] Arntzen, J. W. , Wielstra, B. , & Wallis, G. P. (2014). The modality of nine *Triturus* newt hybrid zones assessed with nuclear, mitochondrial and morphological data. Biological Journal of the Linnean Society, 113, 604–622. 10.1111/bij.12358

[ece35683-bib-0010] Barton, N. H. (1983). Multilocus clines. Evolution, 37, 454–471. 10.1111/j.1558-5646.1983.tb05563.x 28563316

[ece35683-bib-0011] Barton, N. H. (2001). The role of hybridization in evolution. Molecular Ecology, 10, 551–568. 10.1046/j.1365-294x.2001.01216.x 11298968

[ece35683-bib-0012] Barton, N. H. , & Gale, K. S. (1993). Genetic analysis of hybrid zones In Harrison R. G. (Ed.), Hybrid zones and the evolutionary process (pp. 13–45). New York, NY: Oxford University. Press.

[ece35683-bib-0013] Barton, N. H. , & Hewitt, G. M. (1985). Analysis of hybrid zones. Annual Review of Ecology and Systematics, 16, 113–148. 10.1146/annurev.es.16.110185.000553

[ece35683-bib-0014] Blab, J. , & Blab, L. (1981). Quantitative Analysen zur Phänologie, Erfaßbarkeit und Populationsdynamik von Molchbeständen des Kottenforstes bei Bonn. Salamandra, 17, 147–172.

[ece35683-bib-0015] Bock, D. , Hennig, V. , & Steinfartz, S. (2009). The use of fish funnel traps for monitoring crested newts (*Triturus cristatus*) according to the Habitats Directive. Zeitschrift für Feldherpetologie, 15, 1–10.

[ece35683-bib-0016] Brede, E. G. , Thorpe, R. S. , Arntzen, J. W. , & Langton, T. E. S. (2000). A morphometric study of a hybrid newt population (*Triturus cristatus*/*T*. *carnifex*): Beam Brook Nurseries, Surrey, U.K. Biological Journal of the Linnean Society, 70, 685–695. 10.1006/bijl.1999.0423

[ece35683-bib-0017] Callan, H. G. , & Spurway, H. (1951). A study of meiosis in interracial hybrids of the newt, *Triturus cristatus* . Journal of Genetics, 50, 235–249. 10.1007/BF02996220 24539705

[ece35683-bib-0018] Canestrelli, D. , Sacco, F. , & Nascetti, G. (2011). On glacial refugia, genetic diversity, and microevolutionary processes: Deep phylogeographical structure in the endemic newt *Lissotriton italicus* . Biological Journal of the Linnean Society, 105, 42–55. 10.1111/j.1095-8312.2011.01767.x

[ece35683-bib-0019] Cooke, S. D. , Cooke, A. S. , & Sparks, T. H. (1994). Effects of scrub cover on great crested newts breeding performance In Gent T., & Bray R. (Eds.), Conservation and management of great crested newts (pp. 71–74). Peterborough, UK: English Nature.

[ece35683-bib-0020] Coyne, J. A. , & Orr, H. A. (2004). The evolutionary genetics of speciation. Philosophical Transactions of the Royal Society of London. Series B: Biological Sciences, 353, 287–305. 10.1098/rstb.1998.0210 PMC1692208 9533126

[ece35683-bib-0021] Earl, D. A. , & vonHoldt, B. M. (2012). STRUCTURE HARVESTER: A website and program for visualizing STRUCTURE output and implementing the Evanno method. Conservation Genetic Resources, 4, 359–361. 10.1007/s12686-011-9548-7

[ece35683-bib-0022] Evanno, G. , Regnaut, S. , & Goudet, J. (2005). Detecting the number of clusters of individuals using the software STRUCTURE: A simulation study. Molecular Ecology, 14, 2611–2620. 10.1111/j.1365-294X.2005.02553.x 15969739

[ece35683-bib-0023] Fahrbach, M. , & Gerlach, U. (2018). The genus *Triturus*. History, biology, systematics, captive breeding. Frankfurter Beiträge zur Naturkunde (vol. 69). Frankfurt am Main, Germany: Edition Chimaira.

[ece35683-bib-0024] French, N. , Yu, S. , Biggs, P. , Holland, B. , Fearnhead, P. , Binney, B. , … Carter, P. (2014). Evolution of Campylobacter species in New Zealand In Sheppard S. K., & Méric G. (Eds.), Campylobacter ecology and evolution (pp. 221–240). Norfolk, UK: Caister Academic Press.

[ece35683-bib-0025] Guillot, G. , Estoup, A. , Mortier, F. , & Cosson, J. F. (2005). A spatial statistical model for landscape genetics. Genetics, 170, 1261–1280. 10.1534/genetics.104.033803 15520263 PMC1451194

[ece35683-bib-0026] Guillot, G. , Mortier, F. , & Estoup, A. (2005). Geneland: A program for landscape genetics. Molecular Ecology Notes, 5, 712–715. 10.1111/j.1471-8286.2005.01031.x

[ece35683-bib-0027] Hall, T. A. (1999). BioEdit: A user‐friendly biological sequence alignment editor and analysis program for Windows 95/98/NT. Nucleic Acids Symposium Series, 41, 95–98. 10.1021/bk-1999-0734.ch008

[ece35683-bib-0028] Harper, L. R. , Downie, J. R. , & McNeill, D. C. (2018). Assessment of habitat and survey criteria for the great crested newt (*Triturus cristatus*) in Scotland: A case study on a translocated population. Hydrobiologia, 828, 57–71. 10.1007/s10750-018-3796-4

[ece35683-bib-0029] Hewitt, G. M. (2001). Speciation, hybrid zones and phylogeography – or seeing genes in space and time. Molecular Ecology, 10, 537–549. 10.1046/j.1365-294x.2001.01202.x 11298967

[ece35683-bib-0030] Hewitt, G. M. (2011). Quaternary phylogeography: The roots of hybrid zones. Genetica, 139, 617–638. 10.1007/s10709-011-9547-3 21234647

[ece35683-bib-0031] IBM Corp. (2015). IBM SPSS statistics for windows, Version 23.0. Armonk, NY: IBM Corp.

[ece35683-bib-0032] Kalezić, M. L. , Džukić, G. , Stamenković, S. , & Crnobrnja, J. (1990). Morphometrics of the crested newt (*Triturus cristatus* complex) from Yugoslavia: Relevance for taxonomy. Arhiv Bioloških Nauka, 42, 17–37.

[ece35683-bib-0033] Key, K. H. L. (1968). The concept of stasipatric speciation. Systematic Zoology, 17, 14–22. 10.2307/2412391

[ece35683-bib-0034] Klopfstein, S. , Currat, M. , & Excoffier, L. (2006). The fate of mutations surfing on the wave of a range expansion. Molecular Biology and Evolution, 23, 482–490. 10.1093/molbev/msj057 16280540

[ece35683-bib-0035] Krupa, A. P. , Jehle, R. , Dawson, D. A. , Gentle, L. K. , Gibbs, M. , Arntzen, J. W. , & Burke, T. (2002). Microsatellite loci in the crested newt (*Triturus cristatus*) and their utility in other newt taxa. Conservation Genetics, 3, 87–89. 10.1023/A:1014239225553

[ece35683-bib-0036] MacGregor, G. R. , Russell, L. D. , Van Beek, M. E. , Hanten, G. R. , Kovac, M. J. , Kozak, C. A. , … Overbeek, P. A. (1990). Symplastic spermatids (sys): A recessive insertional mutation in mice causing a defect in spermatogenesis. Proceedings of the National Academy of Sciences of the United States America, 87, 5016–5020. 10.1073/pnas.87.13.5016 PMC54252 2164218

[ece35683-bib-0037] Macholán, M. , Munclinger, P. , Šugerková, M. , Dufková, P. , Bímová, B. , Božíková, E. , … Piálek, J. (2007). Genetic analysis of autosomal and X‐linked markers across a mouse hybrid zone. Evolution, 61, 746–771. 10.1111/j.1558-5646.2007.00065.x 17439609

[ece35683-bib-0038] Maletzky, A. , Kyek, M. , & Goldschmid, A. (2007). Monitoring status, habitat features and amphibian species richness of crested newt (*Triturus cristatus* superspecies) ponds at the edge of the species range (Salzburg, Austria). Annales de Limnologie – International Journal of Limnology, 43, 107–115. 10.1051/limn/2007017

[ece35683-bib-0039] Maletzky, A. , Mikulíček, P. , Franzen, M. , Goldschmid, A. , Gruber, H. J. , Horák, A. , & Kyek, M. (2008). Hybridization and introgression between two species of crested newts (*Triturus cristatus* and *T. carnifex*) along contact zones in Germany and Austria: Morphological and molecular data. Herpetology Journal, 18, 1–15.

[ece35683-bib-0040] Meiling, W. R. M. , Arntzen, J. W. , van Delft, J. J. C. V. , & Wielstra, B. (2015). Genetic pollution of a threatened native crested newt species through hybridization with an invasive congener in the Netherlands. Biological Conservation, 184, 145–153. 10.1016/j.biocon.2015.01.022

[ece35683-bib-0041] Mikulíček, P. , Horák, A. , Zavadil, V. , Kautman, J. , & Piálek, J. (2012). Hybridization between three crested newt species (*Triturus cristatus* superspecies) in the Czech Republic and Slovakia: Comparison of nuclear markers and mitochondrial DNA. Folia Zoologica, 61, 202–218. 10.25225/fozo.v61.i3.a4.2012

[ece35683-bib-0042] Mori, E. , Menchetti, M. , Cantini, M. , Bruni, G. , Santini, G. , & Bertolino, S. (2017). Twenty years' monitoring of a population of Italian crested newt *Triturus carnifex*: Strong site fidelity and shifting population structure in response to restoration. Ethology Ecology & Evolution, 29, 460–473.

[ece35683-bib-0043] Neiva, J. , Pearson, G. A. , Valero, M. , & Serrão, E. A. (2010). Surfing the wave on a borrowed board: Range expansion and spread of introgressed organellar genome in the seaweed *Fucus ceranoides* L. Molecular Ecology, 19, 4812–4822. 20958817 10.1111/j.1365-294X.2010.04853.x

[ece35683-bib-0044] Oldham, R. S. , Keeble, J. , Swan, M. J. S. , & Jeffcote, M. (2000). Evaluating the suitability of habitat for the great crested newt (*Triturus cristatus*). Herpetological Journal, 10, 143–155.

[ece35683-bib-0045] Peakall, R. , & Smouse, P. E. (2012). GenAlEx 6.5: Genetic analysis in Excel. Population genetic software for teaching and research – An update. Bioinformatics, 28, 2537–2539. 10.1093/bioinformatics/bts460 22820204 PMC3463245

[ece35683-bib-0046] Piálek, J. , Zavadil, V. , & Valíčková, R. (2000). Morphological evidence for the presence of *Triturus carnifex* in the Czech Republic. Folia Zoologica, 49, 33–40.

[ece35683-bib-0047] Posada, D. , & Crandall, K. A. (2001). Intraspecific gene genealogies: Trees grafting into networks. Trends in Ecology and Evolution, 16, 37–45. 10.1016/S0169-5347(00)02026-7 11146143

[ece35683-bib-0048] Pritchard, J. K. , Stephens, M. , & Donnelly, P. (2000). Inference of population structure using multilocus genotype data. Genetics, 155, 945–959. 10835412 10.1093/genetics/155.2.945 PMC1461096

[ece35683-bib-0049] Reiter, A. , & Hanák, V. (2000). Obojživelníci Národního parku Podyjí. (Amphibians of the Podyjí National Park). Thayensia, 3, 75–146.

[ece35683-bib-0050] Schoorl, J. , & Zuiderwijk, A. (1980). Ecological Isolation in *Triturus cristatus* and *Triturus marmoratus* (Amphibia: Salamandridae). Amphibia‐Reptilia, 1, 235–252. 10.1163/156853881X00357

[ece35683-bib-0051] Shurtliff, Q. R. , Murphy, P. J. , & Matocq, M. D. (2013). Ecological segregation in a small mammal hybrid zone: Habitat‐specific mating opportunities and selection against hybrids restrict gene flow on a fine spatial scale. Evolution, 68, 729–742. 10.1111/evo.12299 24152220

[ece35683-bib-0052] Şova, C. (1973). Morphometric researches in the genus *Triturus* from Romania (Sereth River Basin). Studii Comunicări, Muzeul Ştiinţele Natturii Bacău, 6, 85–286.

[ece35683-bib-0053] Sztatecsny, M. , Jehle, R. , Schmidt, B. R. , & Arntzen, J. W. (2004). The abundance of premetamorphic newts (*Triturus cristatus*, *T. marmoratus*) as a function of habitat determinants: An a priori model selection approach. Herpetological Journal, 15, 89–97.

[ece35683-bib-0054] Szymura, J. M. , & Barton, N. H. (1986). Genetic analysis of a hybrid zone between the fire‐bellied toads, *Bombina bombina* and *B. variegata*, near Cracow in Southern Poland. Evolution, 40, 1141–1159. 28563502 10.1111/j.1558-5646.1986.tb05740.x

[ece35683-bib-0055] Szymura, J. M. , & Barton, N. H. (1991). The genetic structure of the hybrid zone between the fire‐bellied toads *Bombina bombina* and *B. variegat*a: Comparisons between transects and between loci. Evolution, 45, 237–261. 10.1111/j.1558-5646.1991.tb04400.x 28567861

[ece35683-bib-0056] TerBraak, C. J. F. , & Šmilauer, P. (2012). Canoco reference manual and user's guide: Software for ordination, version 5.0. Ithaca, NY: Microcomputer Power.

[ece35683-bib-0057] Thompson, J. S. , Ling, X. , & Grustein, M. (1994). Histone H3 amino terminus is required for telomeric and silent mating locus repression in yeast. Nature, 369, 245–247. 10.1038/369245a0 8183346

[ece35683-bib-0058] Unglaub, B. , Steinfartz, S. , Drechsler, A. , & Schmidt, B. R. (2015). Linking habitat suitability to demography in a pond‐breeding amphibian. Frontiers in Zoology, 12, 9 10.1186/s12983-015-0103-3 25977702 PMC4430901

[ece35683-bib-0059] Visser, M. , Leeuw, M. , Zuiderwijk, A. , & Arntzen, J. W. (2016). Stabilization of a salamander moving hybrid zone. Ecology and Evolution, 7, 689–696. 10.1002/ece3.2676 28116063 PMC5243777

[ece35683-bib-0060] Vörös, J. , & Arntzen, J. W. (2010). Weak population structuring in the Danube crested newt, *Triturus dobrogicus*, inferred from allozymes. Amphibia‐Reptilia, 31, 339–346. 10.1163/156853810791769518

[ece35683-bib-0061] Vörös, J. , Mikulíček, P. , Major, A. , Recuero, E. , & Arntzen, J. W. (2016). Phylogeographic analysis reveals northerly refugia for the riverine amphibian *Triturus dobrogicus* (Caudata: Salamandridae). Biological Journal of the Linnean Society of London, 119, 974–991. 10.1111/bij.12866

[ece35683-bib-0062] Wielstra, B. , & Arntzen, J. W. (2011). Unraveling the rapid radiation of crested newts (*Triturus cristatus* superspecies) using complete mitogenomic sequences. BMC Evolutionary Biology, 11, 62 10.1186/1471-2148-11-162 21672214 PMC3224112

[ece35683-bib-0063] Wielstra, B. , & Artnzen, J. W. (2016). Description of a new species of crested newt, previously subsumed in *Triturus ivanbureschi* (Amphibia: Caudata: Salamandridae). Zootaxa, 4109, 73–80. 10.11646/zootaxa.4109.1.6 27394852

[ece35683-bib-0064] Wielstra, B. , Babik, W. , & Arntzen, J. W. (2015). The crested newt *Triturus cristatus* recolonized temperate Eurasia from an extra‐Mediterranean glacial refugium. Biological Journal of the Linnean Society, 114, 574–587. 10.1111/bij.12446

[ece35683-bib-0065] Wielstra, B. , Burke, T. , Butlin, R. K. , & Arntzen, J. W. (2017). A signature of dynamic biogeography: enclaves indicate past species replacement. Proceedings of the Royal Society B: Biological Sciences, 284(1868), 20172014 10.1098/rspb.2017.2014 PMC5740283 29187631

[ece35683-bib-0066] Wielstra, B. , Burke, T. , Butlin, R. K. , Avcıc, A. , Üzüm, N. , Bozkurt, E. , … Arntzen, J. W. (2017). A genomic footprint of hybrid zone movement in crested newts. Evolution Letters, 1, 93–101. 10.1002/evl3.9 30283642 PMC6121819

[ece35683-bib-0067] Wielstra, B. , McCartney‐Melstad, E. , Arntzen, J. W. , Butlin, R. K. , & Shaffer, H. B. (2019). Phylogenomics of the adaptive radiation of *Triturus* newts supports gradual ecological niche expansion towards an incrementally aquatic lifestyle. Molecular Phylogenetics and Evolution, 133, 120–127. 10.1101/463752 30630099

[ece35683-bib-0068] Wielstra, B. , Sillero, N. , Vörös, J. , & Arntzen, J. W. (2014). The distribution of the crested and marbled newt species (Amphibia: Salamandridae: *Triturus*) – An addition to the New Atlas of Amphibians and Reptiles of Europe. Amphibia‐Reptilia, 35, 376–381. 10.1163/15685381-00002960

[ece35683-bib-0069] Wolterstorff, W. (1923). Uebersicht der Unterarten und Formen des *Triton cristatus* Laur. Blätter für Aquarien und Terrarienkunde, 34, 120–126.

[ece35683-bib-0070] Yanchukov, A. , Hofman, S. , Szymura, J. M. , Mezhzherin, S. V. , Morozov‐Leonov, S. Y. , Barton, N. H. , & Nürnberger, B. (2006). Hybridization of *Bombina bombina* and *B. variegata* (Anura, Discoglossidae) at a sharp ecotone in western Ukraine: Comparisons across transects and over time. Evolution, 60, 583–600. 10.1111/j.0014-3820.2006.tb01139.x 16637503

[ece35683-bib-0071] Zavadil, V. , Piálek, J. , & Klepsch, L. (1994). Extension of the known range of *Triturus dobrogicus*: Electrophoretic and morphological evidence for presence in the Czech Republic. Amphibia‐Reptilia, 15, 329–335. 10.1163/156853894X00362

